# Concomitant Sleep Disorders Significantly Increase the Risk of Cardiovascular Disease in Patients with Psoriasis

**DOI:** 10.1371/journal.pone.0146462

**Published:** 2016-01-08

**Authors:** Hsien-Yi Chiu, Chi-Feng Hsieh, Yi-Ting Chiang, Yi-Wen Tsai, Weng-Foung Huang, Cheng-Yuan Li, Ting-Shun Wang, Tsen-Fang Tsai

**Affiliations:** 1 Institute of Biomedical Engineering, College of Medicine and College of Engineering, National Taiwan University, Taipei, Taiwan; 2 Department of Dermatology, National Taiwan University Hospital Hsin-Chu Branch, Hsinchu, Taiwan; 3 Department of Dermatology, National Taiwan University Hospital and National Taiwan University College of Medicine, Taipei, Taiwan; 4 Institute of Health and Welfare Policy, National Yang-Ming University, Taipei, Taiwan; 5 Department of Dermatology, Taipei Veterans General Hospital, Taoyuan Branch, Taoyuan, Taiwan; 6 Graduate Institute of Clinical Medicine, National Taiwan University College of Medicine, Taipei, Taiwan; 7 Division of Dermatology, National Taiwan University Hospital, Yun-Lin Branch, Dou-Liou, Taiwan; University of Rome Tor Vergata, ITALY

## Abstract

**Background:**

The increased rates of cardiovascular morbidity and mortality in patients with psoriasis are not adequately explained by traditional risk factors. Whether concomitant sleep disorders (SDs) modify the risk of cardiovascular disease (CVD) in patients with psoriasis remains unknown.

**Methods:**

Using the Taiwan National Health Insurance Research Database (NHIRD), we conducted a cohort study to investigate the association between concomitant SDs and CVD risk in patients with psoriasis. Data from 99,628 adults who received a psoriasis diagnosis during the period from 2004 to 2010 were analyzed. Cox proportional hazards regression analysis models were used to compare the risks of ischemic heart disease (IHD) and stroke between patients with and without SDs.

**Results:**

Psoriasis patients with a concomitant SD had significantly higher risks of IHD (adjusted hazard ratio [aHR], 1.25; 95% confidence interval [CI], 1.22–1.28) and stroke (aHR, 1.24; 95% CI, 1.16–1.33) as compared with psoriasis patients without SDs. All psoriasis patient subgroups, including those with mild and severe psoriasis and those with and without arthritis, had increased HRs for IHD and stroke. The increases in IHD and stroke risks conferred by SDs were proportional to the dose of hypnotics used. The effect of SDs on the risks of IHD and stroke was greater in young adults than in middle-aged and older adults.

**Conclusions:**

The risks of IHD and stroke were higher for psoriasis patients with SDs than for those without SDs. Clinicians should carefully evaluate CVD risk, particularly in young patients with psoriasis.

## Introduction

Accumulating evidence suggests that psoriasis, a systemic inflammatory disorder, is associated with a variety of comorbidities, including diabetes, metabolic syndrome, chronic kidney disease, cardiovascular disease (CVD) and cerebrovascular disease [1,2,3,4,5,6,7]. Moreover, psoriasis is associated with pruritus and depression, and pain from psoriatic arthritis may result in difficulty in falling asleep, sleep fragmentation and frequent early awakening [8,9,10]. A case-control study by Wu *et al*. showed that psoriasis is significantly associated with sleep disorders (SDs) and insomnia [11]. A recent study of data from the 2005 National Psoriasis Foundation telephone and e-mail surveys found that 49.5% of respondents reported that their psoriasis adversely affected sleep at least once per month; 11.3% reported that psoriasis interfered with sleep on more than 15 days per month [9]. Poor sleep quality adversely affects quality of life [8]. However, it is not clear whether a concomitant SD modifies CVD risk in patients with psoriasis. Therefore, we conducted a nationwide cohort study to investigate the association between concomitant SDs and CVD risk in patients with psoriasis.

## Materials and Methods

### Dataset

This retrospective cohort study analyzed data from the Taiwan National Health Insurance Research Database (NHIRD), which covered 99% of Taiwan’s population of nearly 23 million people during the period from 2003 to 2011. The NHIRD contains enrollment files and original claims data for reimbursement and includes information on demographic characteristics, International Classification of Diseases, 9th Revision, Clinical Modification (ICD-9-CM) diagnosis and procedure codes, details of prescriptions and comorbidities. This study was approved by the local investigational research bureau of National Taiwan University Hospital HsinChu Branch (HCH 103-024-E). All patient records and information were anonymized and de-identified before the analysis.

### Study population

Using the NHIRD, we selected patients aged 18 years or older who had at least one outpatient visit or admission claim with an ICD-9-CM code for psoriasis (696.0 for psoriatic arthropathy or 696.1 for psoriasis) during 2004–2010. A total of 150,331 individuals were included in the psoriasis patient cohort. The index date was defined as the date of first diagnosis of psoriasis. To identify those SDs that were strongly associated and concomitant with psoriasis, we defined a concomitant SD as any SD that developed within 6 months after a psoriasis diagnosis. Psoriasis patients were excluded if they had received a diagnosis of ischemic heart disease (IHD; ICD-9-CM codes: 410–414), stroke (ICD-9-CM codes: 430–438) or SD (ICD-9-CM codes: 780.52, 307.41, 307.42, 780.51, 780.53 and 780.57) during the period 1 year before the index date, if they had missing data on sex or age or if they were younger than 18 years. We further excluded psoriasis patients who received an SD diagnosis later than 6 months after the index date.

**Fig 1:**
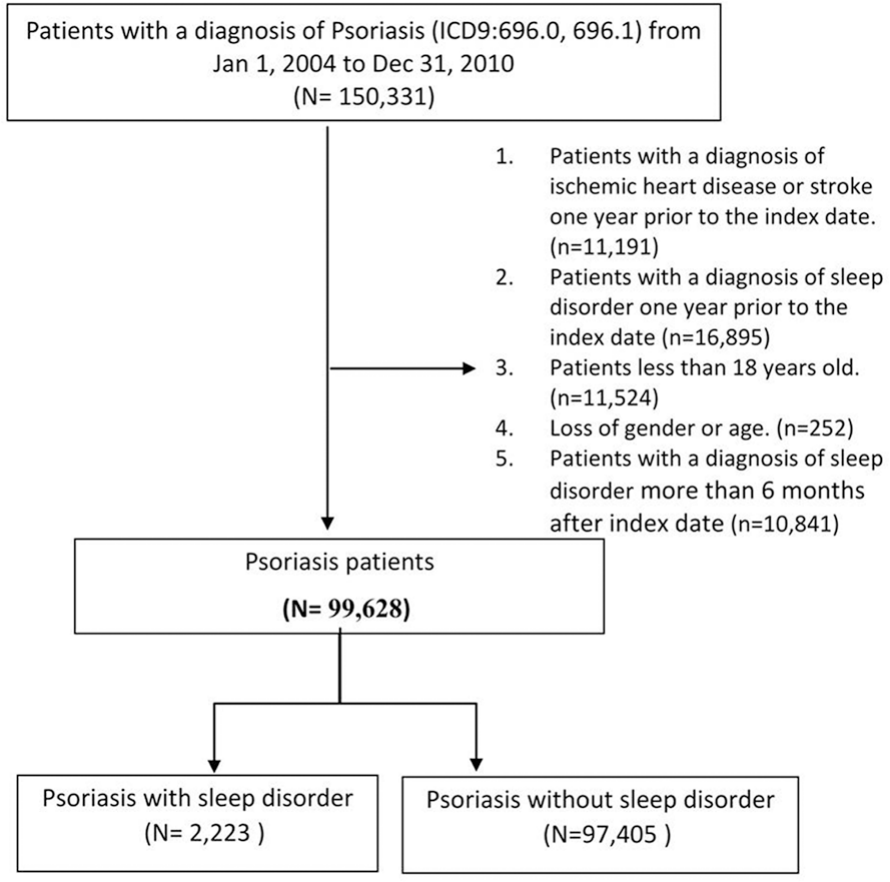
Flowchart. Flowchart of the study subjects.

A total of 99,628 subjects were ultimately included in the analysis and were divided into two groups based on the presence or absence of an SD during the period within 6 months after the index date (with SD, n = 2,223; without SD, n = 97,405). (**Fig 1**). Psoriasis was defined as severe if the patient received systemic antipsoriatic treatment and/or phototherapy (e.g. phototherapy, acitretin, methotrexate, methoxsalen, cyclosporine, azathioprine, hydroxyurea, mycophenolate mofetil, etanercept or adalimumab) and as mild if the patient had not received such treatment. This approach has been used in previous studies. [3,12,13]

### Outcomes and covariates

The primary outcomes in the analysis were CVD, including IHD (ICD-9 codes: 410–414) and stroke (ICD-9 codes: 430–438). The observation period began at the cohort index date and continued until the end of 2011, occurrence of IHD or occurrence of a stroke event. For patients who developed CVD before developing an SD within 6 months after the index date, we assigned the outcomes (including risk-time) before the occurrence of SD from the SD cohort to the reference cohort. We considered and adjusted for a number of covariates, including sex, age, hypertension (ICD-9-CM codes: 401.0, 401.1, 401.9, 402–405, 437.2), diabetes (ICD-9-CM code: 250), hyperlipidemia (ICD-9-CM codes: 272.0, 272.1, 272.2, 272.3, 272.4), obesity (ICD-9-CM codes: 278.0, 278.1) and psychiatric disorders (major depressive disorder, ICD-9-CM codes: 296.2x, 296.3x; minor depression, ICD-9 codes: 311.xx, 300.4x; bipolar disorder, ICD-9 codes: 296.0X, 296.4X, 296.5X, 296.6X, 296.7X, 296.80, 296.89; anxiety, ICD-9 codes: 300.0, 300.2, 300.3, 308.3, 309.81; alcoholism, ICD-9 codes: 303, 305.0) 1 year before the index date, in addition to adjustment for psychiatric disorders within 6 months after the index date.

### Statistical analysis

We used the chi-square test to examine associations between the two SD exposure groups (Y/N) for categorical variables. A Cox proportional hazards model with propensity score weighting was used to estimate hazard ratios (HRs) for the risks of IHD and stroke in patients with and without an SD. We adjusted for potential confounders in the multivariable Cox models. Propensity score weighting was used because we did not want to lose observations of treated subjects and because we wanted an interpretable overall treatment effect. In addition, weighting would allow us to estimate the average treatment effect for the population (ATE weighting) [14]. All statistical tests were two-sided, with an alpha level of 0.05. All confidence intervals (CIs) were 95%. All statistical analyses were performed using SAS version 9.2.

## Results

The 2223 psoriasis patients with an SD were classified as the SD cohort, and the 97,405 psoriasis patients without an SD were the comparison cohort (**Table A in S1 File**). The prevalence of every comorbidity analyzed, including hypertension, diabetes and hyperlipidemia, was higher in psoriasis patients with an SD than in those without an SD (P<0.0001). Psoriasis patients with an SD also had higher incidence rates of IHD (3.91 vs. 2.21 per 100 person-years) and stroke (0.61 vs. 0.34 per 100 person-years) than did psoriasis patients without an SD (**Table 1**). Because patients with psoriasis are more likely to have sleep disturbances, possibly secondary to obesity, depression, anxiety, bipolar disorder and alcoholism, in addition to traditional risk factors for CVD, these potential confounding factors were included and adjusted for in the Cox proportional hazards regression models. Psoriasis with concomitant SD remained associated with higher risks of IHD (adjusted HR [aHR], 1.25; 95% CI, 1.22–1.28) and stroke (aHR, 1.24; 95% CI, 1.16–1.33), as compared with patients without SDs, after adjustment for sex, age category, comorbidities, and propensity score weighting (**Table 2**).

**Table 1:** Incidence rates for ischemic heart disease and stroke in psoriasis patients with and without sleep disorders.

	Total		Psoriasis without sleep disorder		Psoriasis with sleep disorder	
	N	%	N	%	N	%
	99,628	100.00	97,405	97.77	2,223	2.23
Mean duration of follow-up (SD), years	4.39(2.15)		4.39 (2.15)		4.33 (2.23)	
Total follow-up time, person-years	437244.67		427611.59		9633.08	
**All patients with psoriasis**						
Ischemic heart disease or stroke, n (%)	10518	10.56	10116	10.39	402	18.08
Incidence rate*	2.41		2.37		4.17	
Ischemic heart disease, n (%)	9826	9.86	9449	9.70	377	16.96
Incidence rate*	2.25		2.21		3.91	
Stroke, n (%)	1502	1.51	1443	1.48	59	2.65
Incidence rate*	0.34		0.34		0.61	

* per 100 person-years

**Table 2:** Hazard ratios for cardiovascular disease in psoriasis patients with sleep disorders, as compared with psoriasis patients without sleep disorders.

	Crude HR	(95% CI)	P-value	Adjusted HR* (ATE)	(95% CI)	P-value
Ischemic heart disease or acute stroke	1.77	(1.60–1.95)	<0.001	1.38	(1.34–1.41)	<0.001
Ischemic heart disease	1.77	(1.59–1.96)	<0.001	1.25	(1.22–1.28)	<0.001
Stroke	1.74	(1.34–2.25)	<0.001	1.24	(1.16–1.33)	<0.001

Abbreviations: ATE: average treatment effect; HR: hazard ratio; CI: confidence interval; CVD: cardiovascular disease

*Adjusted for sex, age category, comorbidities within 1 year before index date (hypertension, diabetes, hyperlipidemia, obesity, depression, bipolar disorder, anxiety, alcoholism), psychiatric disorders within 6 months after the index date (depression, bipolar disorder, anxiety, alcoholism) and propensity score weighting.

Psoriasis patients were further classified into those with mild or severe psoriasis. In both these subgroups, patients with an SD were more likely than those without an SD to have IHD (**Table 3**). Nevertheless, the aHR was statistically significant only for the mild psoriasis cohort. Regarding cerebrovascular disease, presence of an SD was associated with increased risk for stroke in patients with mild psoriasis (aHR, 1.40; 95% CI, 1.30–1.51). However, in patients with severe psoriasis, the risk for stroke was lower in patients with an SD than in those without an SD, after covariate adjustment. To determine the impact of psoriatic arthritis (PsA) on CVD risk attributable to an SD, we further stratified patients into those with and without PsA. In both these subgroups, patients with an SD had a higher risk than those without an SD of developing IHD (**Table 3**). However, the increased HR for IHD in PsA patients with an SD, as compared with those without an SD, was not significant after covariate adjustment. Although having an SD remained a significant risk factor for stroke in patients without PsA, patients with an SD had a lower risk for stroke than those without an SD in the PsA cohort.

**Table 3:** Cox proportional hazards analysis of CVD risk in subgroups of psoriasis patients with sleep disorders, as compared with psoriasis patients without sleep disorders.

	Crude HR	(95% CI)	P-value	Adjusted HR* (ATE)	(95% CI)	P-value
**Psoriasis with PsA (N = 9,098)**						
Ischemic heart disease or acute stroke	1.57	(1.14–2.16)	0.01	1.12	(1.03–1.22)	0.01
Ischemic heart disease	1.53	(1.10–2.14)	0.01	1.01	(0.93–1.11)	0.76
Stroke	1.32	(0.49–3.59)	0.58	0.64	(0.48–0.84)	<0.01
**Psoriasis without PsA (N = 90,530)**						
Ischemic heart disease or acute stroke	1.79	(1.61–1.99)	<0.001	1.40	(1.37–1.44)	<0.001
Ischemic heart disease	1.79	(1.61–2.00)	<0.001	1.28	(1.24–1.31)	<0.001
Stroke	1.78	(1.36–2.33)	<0.001	1.30	(1.21–1.40)	<0.001
**Severe psoriasis (N = 15,615)**						
Ischemic heart disease or acute stroke	1.34	(1.07–1.69)	0.01	1.12	(1.06–1.19)	<0.001
Ischemic heart disease	1.35	(1.07–1.71)	0.01	1.03	(0.97–1.09)	0.42
Stroke	1.00	(0.49–2.01)	0.99	0.78	(0.66–0.92)	<0.01
**Mild psoriasis (N = 84,013)**						
Ischemic heart disease or acute stroke	1.88	(1.68–2.10)	<0.001	1.44	(1.40–1.48)	<0.001
Ischemic heart disease	1.89	(1.67–2.11)	<0.001	1.32	(1.28–1.36)	<0.001
Stroke	2.06	(1.56–2.73)	<0.001	1.40	(1.30–1.51)	<0.001

Abbreviations: ATE: average treatment effect; HR: hazard ratio; CI: confidence interval

*Adjusted for sex, age category, comorbidities within 1 year before index date (hypertension, diabetes, hyperlipidemia, obesity, depression, bipolar disorder, anxiety, alcoholism), psychiatric disorders within 6 months after the index date (depression, bipolar disorder, anxiety, alcoholism) and propensity score weighting.

We analyzed the risks of IHD and stroke among psoriasis patients with SDs of varying severity. As compared with psoriasis patients without an SD, the aHRs for IHD increased in proportion to hypnotic dose among psoriasis patients with SD (**Table B in S1 File**). We further examined the risks for IHD and stroke in psoriasis patients with non-apneic SDs and apneic SDs (e.g. obstructive sleep apnea disorder). Both non-apneic and apneic SDs were associated with increased risks of IHD and stroke, although the positive association between apneic SDs and CVD was not significant (**Table C in S1 File**).

Age-specific analysis showed that the effect of having an SD on the risks of IHD and stroke decreased with age in psoriasis patients. CVD risk was higher for patients with an SD between the ages of 18 and 34 years than for middle-aged and older adults (attributable risk fractions, 0.69 vs. 0.29 and 0.13, respectively) (**Table D in S1 File**). To confirm the risk of CVD conferred by SDs, we performed sensitivity analysis that excluded patients with CVD comorbidities, including hypertension, lipidemia and diabetes. Psoriasis with SDs remained significantly associated with higher CVD risk (aHR, 1.51; 95% CI, 1.46–1.57; P<0.001), as compared with psoriasis without SDs, which suggests that the primary findings were robust.

## Discussion

Psoriasis is associated with a high CVD burden [5,15]. Cardiovascular death is one of the most common causes of death in patients with psoriasis [12]. However, the increased rates of cardiovascular morbidity and mortality in patients with psoriasis is not adequately explained by traditional risk factors [16]. SDs such as insomnia are a common problem for psoriasis patients, and accumulating evidence from studies of general populations suggest that SDs increase the risks of developing and/or dying from CVD [8]. Pruritus, depression, pain and obstructive sleep apnea, which interferes with the duration and architecture of sleep by increasing nocturnal awakenings, are likely causes of sleep disturbance in patients with psoriasis [8]. Furthermore, the proinflammatory cytokines involved in psoriasis pathogenesis—tumor necrosis factor (TNF) and interleukin (IL)-6—may also alter sleep physiology [17,18,19]. Daytime hypersecretion of these cytokines is associated with poor sleep quality in people with insomnia [18].

SDs, like psoriasis, may predispose patients to CVD risk factors and were found to be positively associated with hypertension, diabetes mellitus, myocardial infarction and stroke in the general population [20,21,22,23,24]. Our results showed that psoriasis patients with an SD had higher risks of hypertension, diabetes mellitus and hyperlipidemia [25,26,27]. However, the exact causes of the association between SDs and CVD remain to be determined. Evidence from epidemiologic and experimental studies suggests that SDs alter inflammatory pathways [28,29,30], endocrine and metabolic profiles [31] and sympathetic nervous activity [32], which results in the development of cardiovascular and cerebrovascular disorders. However, the mechanisms by which SDs affect CVD risk in psoriasis patients have not been thoroughly investigated.

This population-based study found that having an SD significantly increased the risks for IHD and stroke in psoriasis patients, independent of traditional CVD risk factors and psychiatric comorbidities. Accumulating evidence of ongoing systemic inflammation in SDs suggests that such inflammation is a pivotal link to cardiovascular comorbidities [21,22,23,24]. Findings from previous studies suggest that sleep loss increases TNF and IL-6 secretion [18,30]. Using an experimental mouse model, Hirotsu *et al*. showed that sleep deprivation led to significant increases in proinflammatory cytokines (IL-1β, IL-6 and IL-12) and a decrease in an anti-inflammatory cytokine (IL-10), which could exacerbate psoriasis [19]. Moreover, the relationship between inflammation and CVD has long been recognized, and elevated levels of IL-6, TNF-α and CRP are predictors of CVD development [33,34,35]. Thus, SDs may further aggravate systemic inflammation in psoriasis patients, which could trigger or exacerbate CVD.

Greater disease severity and the presence of PsA might increase the inflammatory burden in psoriasis patients [12,36,37,38]. Therefore, we investigated the risk conferred by concomitant SDs in different psoriasis patient subgroups. The HRs for CVD were increased in all subgroups, including those with mild and severe psoriasis and those with and without PsA. The absence of significant associations between presence of an SD and IHD, after adjustment, among psoriasis patients with severe disease and those with PsA might have been due to the higher frequencies of comorbidities in these subgroups, which attenuated the effects of SDs on the risks of IHD and stroke. Moreover, prior studies suggest that systemic methotrexate treatment reduces the incidence of CVD events in psoriasis [39,40]. Thus, use of systemic antipsoriatic therapies as a surrogate marker for severe disease in our study, and the higher proportion of methotrexate use among psoriasis patients with PsA, might have led to an underestimation of CVD risk conferred by having an SD, or even to the presence of an inverse rather than a positive association between SDs and CVD in the severe psoriasis and PsA subgroups.

A previous study reported a positive association between psoriasis and apneic SDs [41], which were found to be associated with CVD and obesity [25,27,41]. The present results indicate that apneic and non-apneic SDs further increase CVD risk for psoriasis patients. The absence of significant associations of apneic SDs with IHD and stroke is likely due to the small size of the apneic SD subgroup. In addition, the age-specific attributable risk fraction showed that the effect of SDs on CVD risk was greater for young adults than for middle-aged and older adults, perhaps because development of additional cardiovascular risk factors coincident with advancing age eventually outweighs the additional CVD risk attributable to SDs in psoriasis patients.

A strength of this study is that it is a large, nationwide, population-based, longitudinal study of the association of SDs with CVD development among psoriasis patients. However, this study has several potential limitations that should be considered. First, selection bias is a concern. The observed associations might reflect healthcare-seeking behavior of individuals with psoriasis, SDs and other comorbidities. The real incidence of SDs may have been underestimated by our use of outpatient and inpatient benefit claims for coding diagnoses. It is possible that some patients included in the control group had SDs for which they did not seek treatment. However, if an association between having an SD and IHD was still observed under these conditions, the true effect would possibly be even greater if data could be collected from “pure” control and case groups. Second, the diagnoses of SDs and psoriasis used in our study relied on administrative claims data, which may be less accurate than diagnoses made in a prospective clinical setting. Patients may be underdiagnosed or overdiagnosed with SDs, resulting in misclassification bias. However, nondifferential misclassification bias would bias the results toward the null. Moreover, previous reports have confirmed the reliability of using the NHIRD in epidemiologic studies of the relationship between insomnia, SDs and CVD [42,43]. Third, defined daily doses of hypnotics were used as a surrogate marker for SD severity in this study. The use and dosage of benzodiazepine hypnotics, however, may not solely depend on SD severity, as other disorders, such as anxiety, may influence benzodiazepine use. Finally, the NHIRD lacked information on the Psoriasis Area and Severity Index, diet, exercise status, and family history of CVD.

Although traditional CVD risk factors are more prevalent among psoriasis patients than among the general population, they do not adequately account for the increased cardiovascular morbidity and mortality in these patients. Our nationwide study showed that concomitant SDs severely impaired quality of life and increased the risk of developing CVD in psoriasis patients, independent of the effects of traditional CVD risk factors. Moreover, the risks of IHD and stroke increased along with the severity of the SDs. The present results highlight the importance of screening for coexisting SDs in psoriasis patients. Therapeutic intervention for SDs may be necessary and could improve cardiovascular health. Psoriasis patients with SDs, particularly young adults, should be carefully evaluated for CVD, regardless of psoriasis severity, the presence of PsA or other metabolic parameters and comorbidities.

## Supporting Information

S1 File Table A. The characteristics of study population. Baseline characteristics of psoriasis patients with and without a sleep disorder. Table B. The dosage of hypnotic drug and CVD risk. The association between the dosage of hypnotic drug use and the risk for CVD among psoriasis patients with a sleep disorder. Table C. The CVD risk in apneic and non-apneic sleep disorder. Cox proportional hazards analysis of the risk for CVD in psoriasis patients with apneic and non-apneic sleep disorder. Table D. The risk of CVD stratified by age. Comparison of the risk of CVD in psoriasis patients with sleep disorder by age.
(DOC)
