# Supplementary material for: Concomitant Sleep Disorders Significantly Increase the Risk of Cardiovascular Disease in Patients with Psoriasis
Source: PLoS One. 2016 Jan 8;11(1):e0146462. doi: 10.1371/journal.pone.0146462 (PMC4712908; doi:10.1371/journal.pone.0146462)
Supplement: S1 File — Comparison of the risk of CVD in psoriasis patients with sleep disorder by age. (DOC) [file pone.0146462.s001.doc]

**Table A.** **The characteristics of study population**. Baseline characteristics of psoriasis patients with and without a sleep disorder.

|  | **Total** | | **Psoriasis without sleep disorder** | | **Psoriasis with sleep disorder** | | P-value |
| --- | --- | --- | --- | --- | --- | --- | --- |
|  | **N** | **%** | **N** | **%** | **N** | **%** |  |
|  | **99,628** | **100.00** | **97,405** | **97.77** | **2,223** | **2.23** |  |
| **Psoriasis Type** |  |  |  |  |  |  |  |
| Psoriatic Arthritis | 4,989 | 5.01 | 4,877 | 5.01 | 112 | 5.04 | <0.001 |
| Psoriasis | 90,530 | 90.87 | 88,548 | 90.91 | 1,982 | 89.16 |  |
| Psoriasis with Psoriatic Arthritis | 4,109 | 4.12 | 3,980 | 4.09 | 129 | 5.80 |  |
| **Age** |  |  |  |  |  |  |  |
| 18-34y | 34,570 | 34.70 | 34,080 | 34.99 | 490 | 22.04 | <0.001 |
| 35-49 | 29,270 | 29.38 | 28,623 | 29.39 | 647 | 29.10 |  |
| 50-64 | 21,428 | 21.51 | 20,849 | 21.40 | 579 | 26.05 |  |
| >=65 y | 14,360 | 14.41 | 13,853 | 14.22 | 507 | 22.81 |  |
| **Sex** |  |  |  |  |  |  |  |
| F | 40,460 | 40.61 | 39,422 | 40.47 | 1,038 | 46.69 | <0.001 |
| M | 59,168 | 59.39 | 57,983 | 59.53 | 1,185 | 53.31 |  |
| **Comorbidity- 1 year prior to index date** |  |  |  |  |  |  |  |
| Hypertension | 15,233 | 15.29 | 14,720 | 15.11 | 513 | 23.08 | <0.001 |
| Diabetes | 8,091 | 8.12 | 7,845 | 8.05 | 246 | 11.07 | <0.01 |
| Hyperlipidemia | 7,778 | 7.81 | 7,513 | 7.71 | 265 | 11.92 | <0.001 |
| Obesity | 340 | 0.34 | 331 | 0.34 | 9 | 0.40 | 0.60 |
| Depression | 667 | 0.67 | 612 | 0.63 | 55 | 2.47 | <0.001 |
| Bipolar | 74 | 0.07 | 68 | 0.07 | 6 | 0.27 | <0.01 |
| Anxiety | 1,697 | 1.70 | 1,597 | 1.64 | 100 | 4.50 | <0.001 |
| Alcoholism | 214 | 0.21 | 198 | 0.20 | 16 | 0.72 | <0.001 |
| **After index date** |  |  |  |  |  |  |  |
| Depression | 423 | 0.42 | 333 | 0.34 | 90 | 4.05 | <0.001 |
| Bipolar | 44 | 0.04 | 35 | 0.04 | 9 | 0.40 | <0.001 |
| Anxiety | 1,024 | 1.03 | 819 | 0.84 | 205 | 9.22 | <0.001 |
| Alcoholism | 116 | 0.12 | 103 | 0.11 | 13 | 0.58 | <0.001 |

**Table B.** **The dosage of hypnotic drug and CVD risk.** The association between the dosage of hypnotic drug use and the risk for CVD among psoriasis patients with a sleep disorder.

|  | **Adjusted HR* (ATE)** | **(95 % CI)** | **P-value** |
| --- | --- | --- | --- |
|  |  |  |  |
| Low | 1.00 |  |  |
| Medium | 1.68 | (1.61-1.75) | <0.001 |
| High | 2.01 | (1.93-2.09) | <0.001 |

Abbreviations: CVD: cardiovascular diseases; HR: hazard ratio; CI: confidence interval

*Adjusted for sex, age categories, comorbidities within 1 year before index date (hypertension, diabetes, hyperlipidemia, obesity, depression, bipolar disorder, anxiety, alcoholism), psychiatric disorders within 6 months after the index date (depression, bipolar disorder, anxiety, alcoholism) and propensity score weighting.

**Table C. The CVD risk in apneic and non-apneic sleep disorder. Cox proportional hazards analysis of the risk for CVD in psoriasis patients with apneic and non-apneic sleep disorder.**

|  | **Crude HR** | **(95 % CI)** | **P-value** | **Adjusted HR* (ATE)** | **(95 % CI)** | **P-value** |
| --- | --- | --- | --- | --- | --- | --- |
| **Apnea (N=97,491)** |  |  |  |  |  |  |
| Ischemic heart disease or acute stroke | 1.29 | (0.72-2.33) | 0.40 | 1.08 | (0.97-1.20) | 0.15 |
| Ischemic heart disease | 1.26 | (0.68-2.34) | 0.47 | 0.90 | (0.80-1.01) | 0.08 |
| Stroke | 1.61 | (0.40-6.40) | 0.50 | 1.26 | (0.98-1.63) | 0.08 |
| **Non-apnea (N=99,397)** |  |  |  |  |  |  |
| Ischemic heart disease or acute stroke | 1.79 | (1.62-1.99) | <0.001 | 1.40 | (1.37-1.44) | <0.001 |
| Ischemic heart disease | 1.80 | (1.62-2.00) | <0.001 | 1.28 | (1.24-1.31) | <0.001 |
| Stroke | 1.68 | (1.28-2.20) | <0.001 | 1.20 | (1.12-1.29) | <0.001 |

Abbreviations: CVD: cardiovascular diseases; HR: hazard ratio; CI: confidence interval

*Adjusted for sex, age categories, comorbidities within 1 year before index date (hypertension, diabetes, hyperlipidemia, obesity, depression, bipolar disorder, anxiety, alcoholism), psychiatric disorder within 6 months after the index date (depression, bipolar disorder, anxiety, alcoholism) and propensity score weighting.

**Table D. The risk of CVD stratified by age.** Comparison of the risk of CVD in psoriasis patients with sleep disorder by age.

|  | **Incidence rate ratio** | **Attributable risk fraction** | **Population attributable fraction (%)** |
| --- | --- | --- | --- |
| **Psoriasis with sleep disorders** |  |  |  |
| 18-34y | 3.18 (2.15-4.71) | 0.69 | 3.07% |
| 35-49 | 1.40 (1.11-1.78) | 0.29 | 0.89% |
| 50-64 | 1.45 (1.22-1.71) | 0.31 | 1.19% |
| >=65 y | 1.15(0.98-1.34) | 0.13 | 0.53% |

Abbreviations: CVD: cardiovascular diseases.
